# Radiosynthesis and in vivo evaluation of ^11^C-labeled BMS-193885 and its desmethyl analog as PET tracers for neuropeptide Y1 receptors

**DOI:** 10.1186/s41181-019-0056-5

**Published:** 2019-02-18

**Authors:** Kazunori Kawamura, Wakana Mori, Masayuki Fujinaga, Tomoteru Yamasaki, Yiding Zhang, Hidekatsu Wakizaka, Akiko Hatori, Lin Xie, Katsushi Kumata, Takayuki Ohkubo, Yusuke Kurihara, Masanao Ogawa, Nobuki Nengaki, Ming-Rong Zhang

**Affiliations:** 1 Department of Radiopharmaceuticals Development, National Institute of Radiological Sciences, National Institutes for Quantum and Radiological Science and Technology, 4-9-1 Anagawa, Inage-ku, Chiba, 263-8555 Japan; 2 Department of Medical Physics, National Institute of Radiological Sciences, National Institutes for Quantum and Radiological Science and Technology, Chiba, 263-8555 Japan; 3 SHI Accelerator Service Ltd, Tokyo, 141-0032 Japan

**Keywords:** Carbon-11, Positron emission tomography, Neuropeptide Y1 receptor, BMS-193885

## Abstract

**Background:**

Neuropeptide Y (NPY) has been implicated in a wide variety of physiological processes, including feeding, learning, memory, emotion, cardiovascular homeostasis, hormone secretion, and circadian rhythms. NPY Yl receptor (NPY Y1-R) is the most widely studied NPY receptor, and is involved in many of these processes. BMS-193885 (**1**) was previously developed as a potent and selective NPY Y1-R antagonist, which has good systemic bioavailability and brain penetration. To evaluate the characteristics of **1** in vivo, we developed ^11^C-labeled BMS-193885 ([^11^C]**1**) and its desmethyl analog ([^11^C]**2**) for potential use as two new positron emission tomography (PET) tracers.

**Results:**

[^11^C]**1** was synthesized from [^11^C]methyl iodide using **2**. [^11^C]**2** was synthesized from [^11^C]phosgene using its aniline and amine derivatives. The mean ± S.D. decay-corrected radiochemical yields of [^11^C]**1** and [^11^C]**2** from ^11^CO_2_ at the end of radionuclide production were 23 ± 3.2% (*n* = 6) and 24 ± 1.5% (*n* = 4), respectively. In biodistribution on mice, radioactivity levels for both tracers were relatively high in the kidney, small intestine, and liver at 60 min post-injection. The radioactivity levels in the kidney, lung, and spleen of mice at 30 min post-injection with [^11^C]**1** were significantly reduced by pretreatment with **1** (10 mg/kg), and levels of [^11^C]**1** in the brain of mice were significantly increased by pretreatment with the P-glycoprotein and breast cancer resistance protein inhibitor elacridar (10 mg/kg). In metabolite analysis using mouse plasma, [^11^C]**1** and [^11^C]**2** were rapidly metabolized within 30 min post-injection, and [^11^C]**1** was mainly metabolized into unlabeled **2** and radiolabeled components.

**Conclusion:**

[^11^C]**1** and [^11^C]**2** were successfully synthesized with sufficient amount of radioactivity and high quality for use in vivo. Our study of [^11^C]**1** and its desmethyl analog [^11^C]**2** was useful in that it helped to elucidate the in vivo characteristics of **1**.

**Electronic supplementary material:**

The online version of this article (10.1186/s41181-019-0056-5) contains supplementary material, which is available to authorized users.

## Background

Neuropeptide Y (NPY) is a 36 amino acid-long peptide that is a member of the pancreatic family of polypeptides and acts as a neurotransmitter or neuromodulator (Tatemoto et al. 1982). NPY is widely distributed in the body, and is an important neuropeptide in both central and peripheral neurons (Tatemoto 1982; Tatemoto et al. 1982; Lundberg et al. 1982). NPY has been implicated in a wide variety of physiological processes, including feeding (Clark et al. 1984; Stanley and Leibowitz 1985; Flood and Morley 1989), learning and memory (Flood and Morley 1989), cardiovascular functions (Jacques et al. 2007), and circadian rhythms (Sindelar et al. 2005). NPY mediates these processes via interactions with a family of at least five G-protein coupled receptors (Y1, Y2, Y4, Y5, and Y6) (Lin et al. 2004). The NPY Y1 receptors (NPY Y1-Rs) consist of 384 amino acids and are pharmacologically distinct from other NPY receptor subtypes (Herzog et al. 1992). NPY Y1-R is the most abundant in the hypothalamus of rats and humans (Jacques et al. 1996; Parker and Herzog 1999), and in the arterioles of various peripheral tissues, such as the thyroid, parathyroid glands, heart, spleen, and digestive systems of rats and mice (Matsuda et al. 2002). A number of peptides and small molecules have been characterized as NPY Y1-R antagonists, and have thus demonstrated clinical potential for use in the treatment of obesity (Yang et al. 2018). To image the function of NPY Y1-Rs in vivo, several radiolabeled tracers have been developed (Langer et al. 2001; Zwanziger et al. 2008; Kameda et al. 2009; Guérin et al. 2010; Zhang et al. 2016; Keller et al. 2017). Recently, a non-peptide ^18^F-labeled NPY Y1-R antagonist ([^18^F]Y1–973) was developed and demonstrated to have effective properties as a positron emission tomography (PET) tracer for imaging NPY Y1-Rs in the central nervous system (Kameda et al. 2009; Hostetler et al. 2011).

**Fig. 1:**
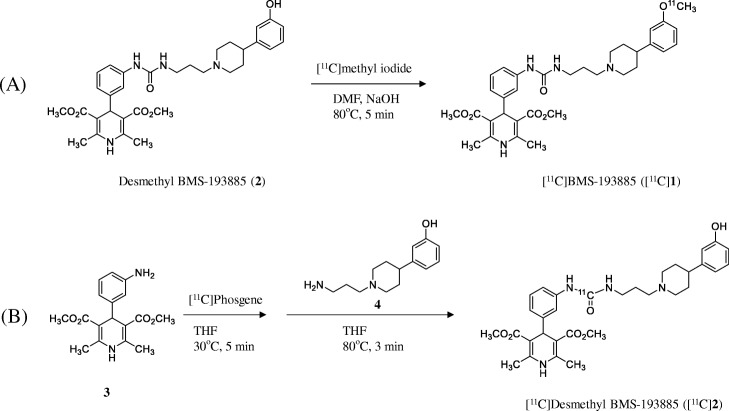
Synthesis of [^11^C]**1** via [^11^C]methyl iodide (A) and [^11^C]**2** via [^11^C]phosgene (B)

BMS-193885 (1,4-dihydro-[3-[[[[3-[4-(3-methoxyphenyl)-1-piperidinyl]propyl]amino]carbonyl]amino]phenyl]-2,6-dimethyl-3,5-pyridinedicarboxylic acid, dimethyl ester; **1**, Fig. 1) was developed as a potent and selective NPY Y1-R antagonist (*K*i = 3.3 nM) (Poindexter et al. 2002) that has good systemic bioavailability and brain penetration, but lacks oral bioavailability (Antal-Zimanyi et al. 2008). Further, **1** also reduced food intake and body weight in animal models of obesity after both acute and chronic administration while inducing no serious adverse cardiovascular effects in rats and dogs (Antal-Zimanyi et al. 2008). Thus, **1** has been determined to be an efficacious pharmacological tool for treating obesity in animal models. Because compound **1** structurally has a methoxy group on the benzene ring, we synthesized [^11^C]**1** by methylation of its desmethyl analog (**2**) with [^11^C]methyl iodide. This radiolabeling does not change the chemical structure and pharmacological profiles of **1**. In addition, we synthesized ^11^C-labeled desmethyl analog of **1** ([^11^C]**2**), because **2** also has high affinity for NPY Y1-R (Ki = 2.7 nM) (Poindexter et al. 2002), and lower lipophilicity (cLogD = 3.3) than **1** (cLogD = 3.8). Compound **2** structurally has an urea moiety, so we performed synthesis of [^11^C]**2** using [^11^C]phosgene as a reactive radiolabeling agent which is routinely produced in our facility. To evaluate and clarify the in vivo characteristic of **1**, such as its biodistribution, metabolism, and the effects of ATP-binding cassette (ABC) transporters on the whole-body uptake of its radioactivity, in this study we evaluated the in vivo characteristics of ^11^C-labeled BMS-193885 ([^11^C]**1**, Fig. 1) and its *O*-desmethyl analog ([^11^C]**2**, Fig. 1).

## Methods

### General

BMS-193885 (**1**, Fig. 1) and elacridar (a P-glycoprotein [P-gp] and breast cancer resistance protein [BCRP] inhibitor) were purchased from Tocris Bioscience (Bristol, UK) or Sigma-Aldrich (St. Louis, MO, USA). Dimethyl 4-(3-(3-(3-(4-(3-hydroxyphenyl)piperidin-1-yl)propyl)ureido)phenyl)-2,6-dimethyl-1,4-dihydropyridine-3,5-dicarboxylate (**2**, Fig. 1), dimethyl 4-(3-isocyanatophenyl)-2,6-dimethyl-1,4-dihydropyridine-3,5-dicarboxylate (**3**, Fig. 1), and 3-(1-(3-aminopropyl)piperidin-4-yl)phenol (**4**, Fig. 1) were synthesized in-house according to procedures reported previously (Poindexter et al. 2002), and were identified by nuclear magnetic resonance and high-resolution mass spectrometry to be consistent with the data described previously (Poindexter et al. 2002). All reagents and organic solvents used were commercially available (Sigma-Aldrich, St. Louis, MO, USA; Tokyo Chemical Industry, Tokyo, Japan; FUJIFILM Wako Pure Chemical, Osaka, Japan) and were used without further purification.

Preparative high-performance liquid chromatography (HPLC) and analytical HPLC were performed using a Jasco HPLC system (with a PU-2089 pump and UV-2070 detector; Jasco, Tokyo, Japan). Effluent radioactivity was monitored using a NaI (Tl) scintillation detector system (OKEN, Tokyo, Japan). HPLC for metabolite analysis was performed using a radio-HPLC system (with a PU-2089 pump, UV-2075 UV detector, and S-2493A radioactivity detector [OKEN]) (Takei et al. 2001). Unless otherwise stated, radioactivity was determined using an IGC-3R or IGC-7F Curiemeter (Hitachi, Tokyo, Japan).

Males from an inbred strain of laboratory mice (ddY, aged 7–8 weeks) were purchased from Japan SLC (Shizuoka, Japan). Animal experiments were approved by the committee for the Care and Use of Laboratory Animals of National Institutes for Quantum and Radiological Science and Technology (Protocol No. 16–1006-6) and performed according to the ARRIVE guidelines (http://www.nc3rs.org.uk/arrive-guidelines).

Lipophilicity (cLog D) was calculated using the ADMET Predictor 8.1 software (Simulations Plus, Lancaster, CA, USA).

### Radiosynthesis of [^11^C]1 from [^11^C]methyl iodide

[^11^C]**1** was synthesized by ^11^C-methylation of **2** using an automated synthesis system developed in-house (Fukumura et al. 2007) (Fig. 1). A solution of **2** (1 mg) and 0.5 mol/L sodium hydroxide solution (3.5 μL) in *N*,*N*-dimethylformamide (DMF) (0.3 mL) was added to a dry septum-equipped vial before radiosynthesis. [^11^C]Methyl iodide was produced by reduction of cyclotron-produced [^11^C]carbon dioxide with lithium aluminum hydride, followed by iodination with hydroiodic acid. The [^11^C]methyl iodide produced was next trapped in the reaction vial containing **2** in DMF. The reaction mixture was heated and held at 80 °C for 5 min. After cooling, 1.0 mL of the preparative HPLC eluent was added to the mixture. The solution was then passed through the preparative HPLC system mentioned above. Preparative HPLC purification was performed on a COSMOSIL PBr column (5 μm mesh, 10 mm inner diameter [i.d.] × 250 mm length; Nacalai Tesque, Kyoto, Japan) using a mixture of acetonitrile and 50 mmol/L ammonium acetate (80:20, vol./vol.) as the mobile phase at a flow rate of 6 mL/min. The retention times of **2** and [^11^C]**1** were approximately 5 min and 10 min, respectively. The HPLC fractions of [^11^C]**1** were collected in flasks to which Tween 80 (75 μL) in ethanol (0.3 mL) had been added before radiosynthesis. The fractions were subsequently evaporated to dryness, and the residues were dissolved in physiological saline.

The product was analyzed by HPLC with radioactivity and ultraviolet detection at 254 nm using a CAPCELL PAK C18 UG80 column (5 μm mesh, 4.6 mm i.d. × 250 mm length; Osaka Soda, Osaka, Japan). Elution was performed using a mixture of acetonitrile, water, and triethylamine (60:40:0.1, vol./vol./vol.) at a flow rate of 1.0 mL/min. The retention time of [^11^C]**1** was 7.5 min (Additional file 1: Figures S1-1 and S1-2).

### Radiosynthesis of [^11^C]2 from [^11^C]phosgene

[^11^C]Phosgene was synthesized by the initial production of [^11^C]carbon dioxide using a cyclotron (CYPRIS HM-18; Sumitomo Heavy Industries, Tokyo, Japan) and an automated synthesis unit as described previously (Ogawa et al. 2010). Reduction of [^11^C]carbon dioxide with H_2_ in the presence of a nickel catalyst produced [^11^C]methane, and this step was then followed by the chlorination of [^11^C]methane and conversion of [^11^C]carbon tetrachloride to [^11^C]phosgene (Ogawa et al. 2010). [^11^C]**2** was synthesized from [^11^C]phosgene in the following manner (Fig. 1). Aniline derivative (**3**, 1.0 mg) in tetrahydrofuran (THF) solution (0.2 mL) was added to a dry septum-equipped vial just before radiosynthesis. [^11^C] Phosgene was trapped in the solution containing **3** at − 15 °C for 1 min. The reaction mixture was heated at 30 °C for 3 min. After cooling, a solution of amine derivative (**4**, 1.0 mg) in THF (0.2 mL) was added to the reaction mixture of **3**. The reaction mixture was heated at 80 °C for 3 min to remove THF. Then, 0.5 mL of the preparative HPLC eluent was added to the reaction mixture. The solution was then passed through the preparative HPLC system mentioned above. Preparative HPLC purification was performed on a CAPCELL PAK C18 UG80 column (5 μm mesh, 10 mm i.d. × 250 mm length) using a mixture of acetonitrile and 50 mmol/L ammonium acetate (40:60, vol./vol.) as the mobile phase at a flow rate of 5 mL/min. The retention time of [^11^C]**2** was approximately 7 min. The HPLC fractions of [^11^C]**2** were collected in flasks to which Tween 80 (75 μL) in ethanol (0.3 mL) had been added before radiosynthesis. The fractions were subsequently evaporated to dryness, and the residues were dissolved in physiological saline. The product was analyzed by HPLC as mentioned above. The retention time of [^11^C]**2** was 7.3 min (Additional file 1: Figures S2-1 and S2-2).

### Biodistribution in mice

[^11^C]**1** (13 MBq/0.12 nmol) or [^11^C]**2** (15 MBq/0.36 nmol) was intravenously injected into mice (aged 7–8 weeks, weighing 33–38 g, *n* = 4). Mice were euthanized by cervical dislocation at 5, 15, 30, or 60 min post-injection.

The effects of pretreatment with **1** (BMS-193885) or the ABC transporter-inhibitor elacridar (inhibiting drug efflux transporters, mainly P-gp and BCRP) on tissue distributions of radioactivity were investigated. [^11^C]**1** (7.1–14 MBq/0.079–0.23 nmol) or [^11^C]**2** (6.0 MBq/0.16 nmol) was intravenously injected into mice (aged 7–8 weeks, weighing 32–36 g, *n* = 4–8) 20–30 min after the administration of the solution of **1** (10 mg/kg body weight [b.w.]/0.1 mL water containing 20% Tween 80), elacridar (5 mg/kg b.w./0.1 mL water), or a control fluid (0.1 mL water). Mice were euthanized by cervical dislocation at 30 min post-injection.

Blood samples were collected by heart puncture. Tissues were dissected and weighed. The radioactivity in samples was measured with an automatic gamma counter (Wizard 3′′ 1480, PerkinElmer, Waltham, MA, USA). The tissue uptake of radioactivity was expressed as the percentage of injected dose detected per gram of sample (%ID/g, tissue radioactivity/g tissue/injected radioactivity × 100).

### Analysis of metabolites in mouse plasma

[^11^C]**1** (37–56 MBq/0.73–0.95 nmol) or [^11^C]**2** (46–61 MBq/1.1–1.5 nmol) was intravenously injected into mice (aged 7–9 weeks, weighing 33–43 g, *n* = 4). Mice were euthanized by cervical dislocation at 30 min post-injection. Blood samples taken by heart puncture were collected into heparinized syringes. Samples were centrifuged at 13,000×*g* (Model 5500, KUBOTA, Tokyo, Japan) for 3 min at 4 °C to obtain plasma (0.1–0.2 mL). Plasma samples were added to equivalent volumes of 1 mol/L ammonium acetate solution. Samples were directly loaded into the injector loop, and analyzed using the combination of column-switching HPLC and on-line solid-phase extraction as a modification of the previously described procedures (Chitneni et al. 2008; Gillings 2009; Greuter et al. 2005; Hilton et al. 2000; Kawamura et al. 2013). HPLC analysis was performed using the radio-HPLC system for metabolite analysis mentioned in the ‘General’ section above, and also using a Cadenza HS-C18 column (3 μm mesh, 10 mm i.d. × 150 mm length; Imtakt, Kyoto, Japan) fitted with a Cadenza HS-C18 guard cartridge (3 μm mesh, 10 mm i.d. × 8 mm length; Imtakt). Elution was performed using a 0.1 mol/L ammonium acetate solution for 3 min after loading, a mixture of 90% acetonitrile solution and water (0:100 to 40:60, vol./vol.) from 3 to 4 min after loading, and then a mixture of 90% acetonitrile solution and water (40:60, vol./vol.) from 4 to 12 min after loading. The flow rate was 4 mL/min. The retention times of [^11^C]**1** and [^11^C]**2** were 10.0 and 10.5 min, respectively.

In addition, the effects of co-injection of [^11^C]**1** with **1** on the results of metabolite analyses of plasma were investigated. [^11^C]**1** (26–30 MBq/0.38–0.45 nmol) and a solution of **1** (50 mg/kg b.w. in water containing 20% Tween 80) were intravenously co-injected into mice (aged 7–9 weeks, weighing 35–39 g, *n* = 4). Mice were euthanized by cervical dislocation at 30 min post-injection. Blood samples taken by heart puncture were collected into heparinized syringes. The samples were centrifuged at 13,000×*g* (Model 5500, KUBOTA, Tokyo, Japan) for 3 min at 4 °C to obtain plasma (0.2 mL). Plasma samples were added to equivalent volumes of acetonitrile, and mixtures were centrifuged at 20,000×*g* for 2 min. The precipitate was added to 0.2 mL of acetonitrile, and this mixture was centrifuged at 20,000×*g* for 2 min to obtain the supernatant. The mixed supernatant was added to 0.2 mL of water, and this solution was loaded into the injector loop. HPLC analysis was performed using the abovementioned radio-HPLC system for metabolite analysis, and also a YMC-Triart C18 ExRs column (5 μm mesh, 10 mm i.d. × 150 mm length; YMC, Kyoto, Japan). Elution was performed using a mixture of 90% acetonitrile solution and 0.1 mol/L ammonium acetate solution (45:55, vol./vol.). The flow rate was 4 mL/min. The retention times of **2** and [^11^C]**1** were 3.6 and 7.5 min, respectively.

### Statistical analyses

Quantitative data are expressed herein as mean ± standard deviation (S.D.) values. Differences between control mice and **1** or elacridar-treated mice were examined using one-way analysis of variance (ANOVA), and were considered significant at *p <* 0.05. The data were analyzed using the SigmaPlot 14.0 software package (Systat Software, San Jose, CA, USA).

## Results

### Radiosynthesis of [^11^C]1 from [^11^C]methyl iodide

[^11^C]**1** was synthesized approximately 30 min after the end of irradiation (EOI). The radiochemical yield of [^11^C]**1** from [^11^C]CO_2_ was 23 ± 3.2% at EOI (*n* = 6), molar activity was 87 ± 28 GBq/μmol at the end of synthesis (EOS) (*n* = 6), and radiochemical purity was > 99%. The radioactivity and quality of [^11^C]**1** were sufficient for application to in vivo studies.

### Radiosynthesis of [^11^C]2 from [^11^C]phosgene

[^11^C]**2** was synthesized approximately 33 min after EOI. The radiochemical yield of [^11^C]**2** from [^11^C]CO_2_ was 24 ± 1.5% at EOI (*n* = 4), molar activity was 52 ± 10 GBq/μmol at EOS (*n* = 4), and radiochemical purity was > 99%. The radioactivity and quality of [^11^C]**2** were sufficient for application to in vivo animal studies.

### Biodistribution in mice

The biodistribution of radioactivity in mice after injections of [^11^C]**1** or [^11^C]**2** is summarized in Table 1. After the injection of [^11^C]**1**, the mean radioactivity levels in the blood, heart, lung, liver, spleen, and muscle gradually decreased for 60 min post-injection. In the kidney, the mean radioactivity level after the injection of [^11^C]**1** gradually decreased until 30 min post-injection, and then remained constant up to 60 min post-injection. In the small intestine, the mean radioactivity level gradually increased for 60 min post-injection. In the brain, the mean radioactivity level gradually increased until 15 min had passed, and then remained constant from 15 to 60 min post-injection. At 60 min post-injection with [^11^C]**1**, the radioactivity levels were relatively high in the kidney and small intestine, and moderate in the lung, liver, and pancreas. After the injection with [^11^C]**2**, the mean radioactivity levels in the blood, heart, lung, liver, spleen, and kidney gradually decreased for 60 min post-injection. In the small intestine and pancreas, the mean radioactivity levels gradually increased until 15 min had passed, and then gradually decreased until 60 min post-injection. In muscle and brain tissues, the mean radioactivity levels remained constant for 60 min post-injection. For 60 min post-injection with [^11^C]**2**, radioactivity levels were relatively high in the small intestine, liver, and kidney, and moderate in the pancreas.

**Table 1:** Biodistribution of radioactivity in mice after the injection with [^11^C]**1** or [^11^C]**2**

Tissue	Radioactivity level (%ID/g)^a^							
	5 min^b^		15 min^b^		30 min^b^		60 min^b^	
	[^11^C]**1**	[^11^C]**2**	[^11^C]**1**	[^11^C]**2**	[^11^C]**1**	[^11^C]**2**	[^11^C]**1**	[^11^C]**2**
Blood	0.76±0.07	0.65±0.05	0.60±0.06	0.21±0.01	0.50±0.04	0.17±0.02	0.46±0.06	0.19±0.20
Heart	1.93±0.23	0.69±0.09	1.38±0.04	0.42±0.02	1.10±0.05	0.28±0.04	0.82±0.07	0.14±0.04
Lung	5.08±1.12	1.00±0.08	3.43±0.28	0.79±0.11	2.72±0.21	0.82±0.13	2.13±0.36	0.65±0.08
Liver	16.09±1.85	29.54±1.57	8.82±2.28	24.28±3.04	5.05±0.40	15.54±4.41	4.09±1.60	8.43±2.71
Pancreas	3.40±0.46	1.20±0.18	3.75±0.35	1.27±0.11	3.64±0.22	1.18±0.18	3.70±0.41	1.15±0.13
Spleen	3.18±0.67	0.79±0.11	2.05±0.17	0.55±0.07	1.66±0.04	0.37±0.07	1.41±0.24	0.23±0.05
Kidney	17.41±1.12	13.25±1.18	14.06±1.00	10.96±0.51	12.93±1.01	9.89±0.52	13.37±2.72	9.23±1.61
Small intestine	6.98±2.37	15.78±6.51	9.11±1.31	30.77±8.94	11.02±1.44	27.23±12.69	12.43±1.54	13.52±4.25
Muscle	0.64±0.05	0.44±0.21	0.57±0.08	0.30±0.02	0.52±0.11	0.38±0.17	0.47±0.07	0.33±0.19
Brain	0.21±0.01	0.05±0.02	0.40±0.06	0.04±0.04	0.32±0.05	0.01±0.00	0.32±0.05	0.03±0.01

^a^Mean±S.D. (n=4)

^b^Time elapsed after the injection of ^11^C-labeled tracer

In the heart, lung, spleen, kidney, muscle, and brain, which express NPY Y1 at high levels (Nakamura et al. 1995), the radioactivity levels at 30 and 60 min post-injection with [^11^C]**1** were higher than those post-injection with [^11^C]**2**. In the blood, the radioactivity levels at 30 and 60 min post-injection with [^11^C]**1** were also higher than those post-injection with [^11^C]**2**. In the liver, the radioactivity levels at 30 and 60 min post-injection with [^11^C]**1** were lower than those post-injection with [^11^C]**2**.

**Fig. 2:**
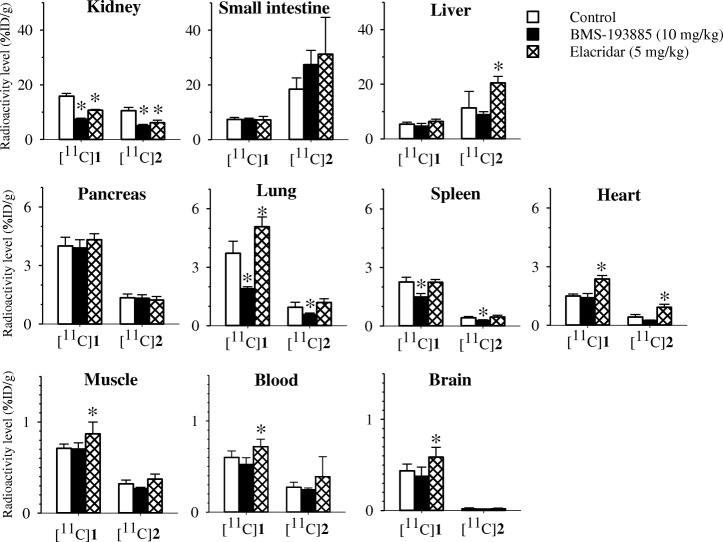
The effects of pretreatment with **1** (10 mg/kg) or elacridar (5 mg/kg) on the biodistribution of radioactivity at 30 min post-injection with [^11^C]**1** or [^11^C]**2** in mice. The injected dose of [^11^C]**1** or [^11^C]**2** were 7.1–14 MBq (0.079–0.23 nmol) or 6.0 MBq (0.16 nmol), respectively. The radioactivity level was expressed as the mean %ID/g

To evaluate the effects of unlabeled **1** or the drug efflux transporter (P-gp and BCRP) inhibitor elacridar on uptake, we performed experiments using a pretreatment of a 10 mg/kg dose of **1** or a 5 mg/kg dose of elacridar administered 20 to 30 min prior to the injection with [^11^C]**1** or [^11^C]**2** (Fig. 2). The mean radioactivity levels in the lung, spleen, and kidney at 30 min post-injection with each tracer were significantly decreased by pretreatment with **1**. In contrast, radioactivity levels in the blood, heart, pancreas, muscle, and brain were not changed by pretreatment with **1**. The mean radioactivity levels in the blood, heart, lung, muscle, and brain at 30 min post-injection with [^11^C]**1** were significantly increased by pretreatment with elacridar. The mean radioactivity levels in the liver and heart at 30 min post-injection of [^11^C]**2** were also significantly increased by pretreatment with elacridar. In contrast, the mean radioactivity levels in the kidney at 30 min post-injection with each tracer were significantly decreased by pretreatment with elacridar.

### Analysis of metabolites in mouse plasma

**Fig. 3:**
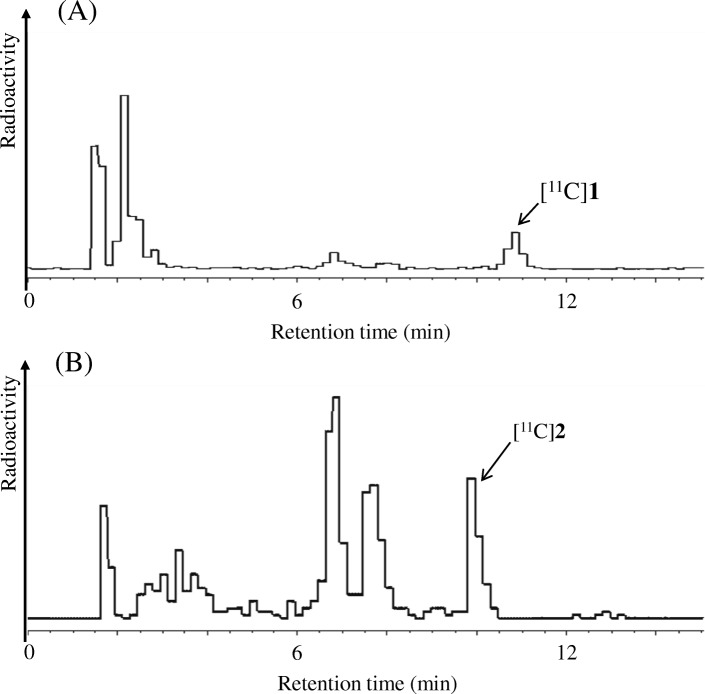
Radio-HPLC chromatograms from metabolite analyses of mouse plasma at 30 min post-injection with [^11^C]**1** (A) or [^11^C]**2** (B). The injected dose of [^11^C]**1** or [^11^C]**2** were 37–56 MBq (0.73–0.95 nmol) or 46–61 MBq (1.1–1.5 nmol), respectively

To examine the percentages of unchanged radiolabeled analytes in the plasma of mice following the injection of [^11^C]**1** or [^11^C]**2**, we performed metabolite analyses using radio-HPLC. The percentages of intact tracer [^11^C]**1** and [^11^C]**2** at 30 min post-injection in the plasma of mice were 11.8 ± 2.5% (Fig. 3a) and 19.9 ± 2.4% (Fig. 3b), respectively.

**Fig. 4:**
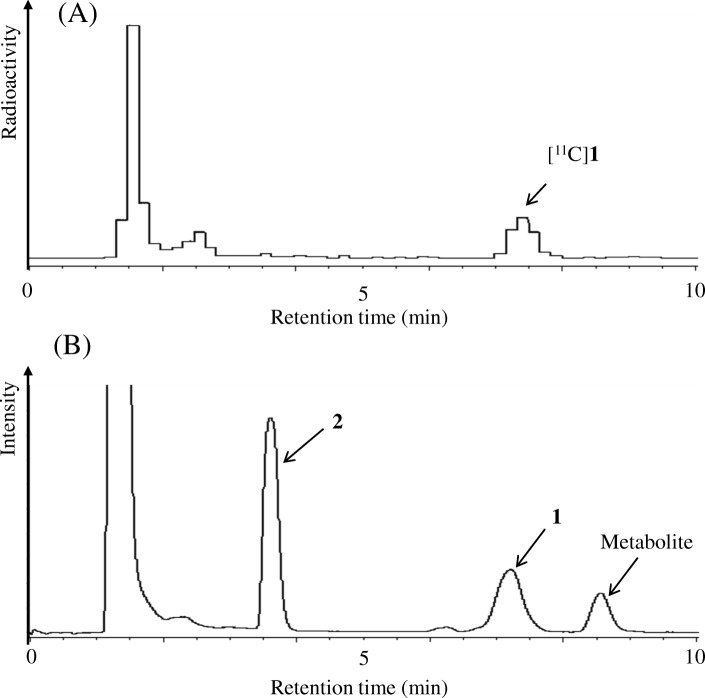
Radio- (A) and UV-HPLC (B) chromatograms from metabolite analyses of mouse plasma at 30 min post-injection with [^11^C]**1** and **1** (50 mg/kg). The injected dose of [^11^C]**1** was 26–30 MBq (0.38–0.45 nmol)

We also performed metabolite analyses to confirm the identities of metabolites present in the plasma at 30 min post-injection with [^11^C]**1** and non-radiolabeled **1**. As shown in Fig. 4, the UV peak corresponding to **2** (retention time = 3.5 min) was the highest among all the detected UV peaks except for that corresponding to the solvent front (retention time = 1.5 min). UV peaks corresponding to **1** and an unknown metabolite were also detected, and presented peaks with moderate areas of UV absorbance (Fig. 4).

## Discussion

We successfully synthesized [^11^C]**1** and [^11^C]**2** with sufficient amount of radioactivity and high quality for applied use as PET tracers for in vivo studies. Synthesis of [^11^C]**2** was easily achieved because [^11^C]phosgene is routinely produced in our facility, and two of its precursors, **3** and **4,** were used for labeling **2** and also used for synthesizing **2** itself for labeling [^11^C]**1**. To determine the in vivo characteristics of **1**, we performed in vivo experiments on mice using [^11^C]**1** and [^11^C]**2**, two radiolabeled analogs of **1**.

In the brain, the radioactivity level after the injection of [^11^C]**1** was relatively low (maximum of 0.4%ID/g at 15 min post-injection). To increase brain penetration in vivo, we developed and evaluated a ^11^C-labeled desmethyl analog of **1** ([^11^C]**2**), which had lower lipophilicity (cLogD = 3.3) than **1** (cLogD = 3.8) and higher binding affinity (*K*i = 2.7 nM) for NPY Y1-R than **1** (*K*i = 3.3 nM) (Poindexter et al. 2002). However, the radioactivity level in the brain at 30 min post-injection with [^11^C]**2** (0.01%ID/g) was much lower than that with [^11^C]**1** (0.32%ID/g). This result showed that [^11^C]**2** penetrated the brain poorly. Therefore, we subsequently assumed that **1** was a P-gp and BCRP substrate or inhibitor that prevents its appropriate absorption into the brain, and then evaluated whether the biodistribution of [^11^C]**1** and [^11^C]**2** in mice was affected by drug efflux transporters using the P-gp and BCRP inhibitor elacridar. As shown in Fig. 2, the radioactivity level in the brain at 30 min post-injection with [^11^C]**1** was significantly increased by pretreatment with elacridar, suggesting that brain penetration of [^11^C]**1** was affected by P-gp and BCRP. In contrast, the radioactivity level in the NPY Y1-R-rich brain at 30 min post-injection with [^11^C]**1** was not decreased by pretreatment with **1**. Therefore, the blockade effect of pretreatment with **1** may be limited due to the effects of drug efflux by P-gp and BCRP. On the other hand, the radioactivity level in the brain at 30 min post-injection with [^11^C]**2** was not increased by pretreatment with elacridar. Meanwhile, the radioactivity levels in the liver and small intestine at 30 min post-injection with [^11^C]**2** was higher than those post-injection with [^11^C]**1**. These results suggested that [^11^C]**2** was metabolized more rapidly than [^11^C]**1** because [^11^C]**2** was accumulated at relatively high levels in the small intestine and liver, which are organs involved in metabolism. Furthermore, in the lung, spleen, and kidney, which are rich in NPY Y1-Rs (Nakamura et al. 1995), the radioactivity levels at 30 min post-injection with [^11^C]**1** or [^11^C]**2** were decreased significantly by pretreatment with **1**. Therefore, we concluded that [^11^C]**1** and [^11^C]**2** may be useful PET tracers for the imaging of NPY Y1-Rs in these peripheral organs. To visualize NPY Y1-Rs in the brain, we are further developing a PET tracer with superior BBB penetration and in vivo stability.

Previous studies reported that unlabeled **1** has good stability in rat liver microsomes in vitro (Poindexter et al. 2002). In the present in vivo study, [^11^C]**1** was rapidly metabolized within 30 min post-injection. Differences between in vitro and in vivo studies’ results may be explained by the rapid metabolism of **1** or [^11^C]**1** in the kidney. In the kidney, the radioactivity level at 60 min post-injection with [^11^C]**1** was the highest among those in all of the investigated organs. Metabolism of drugs in the kidney has been described extensively, and numerous enzymes play a role in the metabolism and clearance of drugs there (Lash 1994; Bajaj et al. 2018). Consequently, the metabolism of [^11^C]**1** in vivo may be faster than that observed in liver microsomes in vitro because [^11^C]**1** is most likely extensively metabolized by the kidney.

## Conclusion

[^11^C]**1** and [^11^C]**2** were successfully synthesized with sufficient amount of radioactivity and high quality for the application of both as PET tracers. We found that [^11^C]**1** was rapidly metabolized into its unlabeled desmethyl analog **2** and radiolabeled components. In NPY Y1-R-rich organs, such as the lung, spleen, and kidney, the radioactivity levels after the injection of [^11^C]**1** and [^11^C]**2** were decreased significantly by the blockade effects of pretreatment with **1**. Our study of [^11^C]**1** and its desmethyl analog [^11^C]**2** was useful in that it helped to elucidate the in vivo characteristics of **1**.

## Additional file

Additional file 1: **Figure S1-1.** The HPLC chromatograms of **1**; **Figure S1-2.** The HPLC chromatograms of [^11^C]**1**; **Figure S2-1.** The HPLC chromatograms of **2**; **Figure S2-2.** The HPLC chromatograms of [^11^C]**2**. (DOCX 124 kb)

## Abbreviations

%ID/g: Percentage of injected dose detected per gram of sample
ABC: ATP-binding cassette
ANOVA: One-way analysis of variance
b.w.: Body weight
BCRP: Breast cancer resistance protein
DMF: *N*,*N*-Dimethylformamide
EOI: End of irradiation
EOS: End of synthesis
HPLC: High-performance liquid chromatography
NPY: Neuropeptide Y
NPY-1R: Neuropeptide Yl receptor
PET: Positron emission tomography
P-gp: P-glycoprotein
S.D.: Standard deviation
