# Supplementary material for: Radiosynthesis and in vivo evaluation of 11C-labeled BMS-193885 and its desmethyl analog as PET tracers for neuropeptide Y1 receptors
Source: EJNMMI Radiopharm Chem. 2019 Feb 18;4:4. doi: 10.1186/s41181-019-0056-5 (PMC6379498; doi:10.1186/s41181-019-0056-5)
Supplement: Supplementary file 1 — Figure S1-1. The HPLC chromatograms of 1; Figure S1-2. The HPLC chromatograms of [11C]1; Figure S2-1. The HPLC chromatograms of 2; Figure S2-2. The HPLC chromatograms of [11C]2. (DOCX 124 kb) [file 41181_2019_56_MOESM1_ESM.docx]

**Supplementary information for:**

**Radiosynthesis and in vivo evaluation of ^11^C-labeled BMS-193885 and its desmethyl analog as PET tracers for neuropeptide Y1 receptors**

Kazunori Kawamura^1^*, Wakana Mori^1^, Masayuki Fujinaga^1^, Tomoteru Yamasaki^1^, Yiding Zhang^1^, Hidekatsu Wakizaka^2^, Akiko Hatori^1^, Lin Xie^1^, Katsushi Kumata^1^, Takayuki Ohkubo^1,3^, Yusuke Kurihara^1,3^, Masanao Ogawa^1,3^, Nobuki Nengaki^1,3^ and Ming-Rong Zhang^1^

^1^Department of Radiopharmaceuticals Development, National Institute of Radiological Sciences, National Institutes for Quantum and Radiological Science and Technology, Chiba 263-8555, Japan

^2^Department of Medical Physics, National Institute of Radiological Sciences, National Institutes for Quantum and Radiological Science and Technology, Chiba 263-8555, Japan

^3^SHI Accelerator Service Ltd., Tokyo 141-0032, Japan

*Correspondence: kawamura.kazunori@qst.go.jp

Department of Radiopharmaceuticals Development, National Institute of Radiological Sciences, National Institutes for Quantum and Radiological Science and Technology, 4-9-1 Anagawa, Inage-ku, Chiba 263-8555, Japan

1. HPLC chromatograms of **1** and [^11^C]**1**

Figure S1-1. The HPLC chromatogram of **1**. Compound **1** was analyzed by HPLC with ultraviolet detection at 254 nm using a CAPCELL PAK C18 UG80 column (5 μm mesh, 4.6 mm i.d. × 250 mm length; Osaka Soda, Osaka, Japan). Elution was performed using a mixture of acetonitrile, water, and triethylamine (60:40:0.1, vol./vol./vol.) at a flow rate of 1.0 mL/min.

Radioactivity

UV

Figure S1-2. The HPLC chromatogram of [^11^C]**1**. The [^11^C]**1** was analyzed by HPLC with radioactivity and ultraviolet detection at 254 nm using a CAPCELL PAK C18 UG80 column (5 μm mesh, 4.6 mm i.d. × 250 mm length; Osaka Soda). Elution was performed using a mixture of acetonitrile, water, and triethylamine (60:40:0.1, vol./vol./vol.) at a flow rate of 1.0 mL/min. The retention time of [^11^C]**1** was 7.5 min.

2. HPLC chromatograms of **2** and [^11^C]**2**

Figure S2-1. The HPLC chromatogram of **2**. Compound **2** was analyzed by HPLC with ultraviolet detection at 254 nm using a CAPCELL PAK C18 UG80 column (5 μm mesh, 4.6 mm i.d. × 250 mm length; Osaka Soda). Elution was performed using a mixture of acetonitrile, water, and triethylamine (60:40:0.1, vol./vol./vol.) at a flow rate of 1.0 mL/min.

UV

Radioactivity

Figure S2-2. The HPLC chromatogram of [^11^C]**2**. The [^11^C]**2** was analyzed by HPLC with radioactivity and ultraviolet detection at 254 nm using a CAPCELL PAK C18 UG80 column (5 μm mesh, 4.6 mm i.d. × 250 mm length; Osaka Soda). Elution was performed using a mixture of acetonitrile, water, and triethylamine (60:40:0.1, vol./vol./vol.) at a flow rate of 1.0 mL/min. The retention time of [^11^C]**2** was 7.3 min.
